# Antimicrobial sensitivity patterns of *Staphylococcus* species isolated from mobile phones and implications in the health sector

**DOI:** 10.1186/s13104-020-05413-7

**Published:** 2021-01-06

**Authors:** Clement Shiluli, Caroly Achok, Philip Nyaswa, Susan Ogwai, Arthur Aroko, James Obila, George Koigi, Mustafa Ridhwana, Bildad Okwayo, Dorcas Wanjiru, Linda Lukeba, Eline Ryckaert, Arne Van Durme, Verena Walschaerts, Vicky De Preter

**Affiliations:** 1 Department of Microbiology, UZIMA University, Kisumu, Kenya; 2 Department of Health, University Colleges Leuven-Limburg (UCLL), Leuven, Belgium

**Keywords:** *Staphylococcus* species, Gram-positive bacteria, Drug-resistant bacteria

## Abstract

**Objectives:**

The aim of this research was to determine drug sensitivity profiles of *Staphylococcus* species isolated from mobile phones of students in Microbiology and Biomedical Laboratory Sciences from UZIMA University, Kisumu (Kenya) and the University Colleges Leuven-Limburg, Leuven (Belgium), respectively.

**Results:**

All mobile phones (16/16, 100%) had gram-positive bacteria. 3/8 (37.5%) mobile devices had *Staphylococcus aureus*. 2/3 (67%) *Staphylococcus aureus* strains were resistant to ampicillin, oxacillin, ceftazidime, vancomycin and amoxicillin. Guidelines for disinfection of mobile phones need to be developed urgently to stop transmission of resistant bacteria.

## Introduction

In 2019, approximately 5 billion people had mobile phones worldwide [1]. Health care workers (HCW) use mobile phones to share X-rays, laboratory reports and electrocardiograms. This improves the quality of care particularly during emergencies [2, 3]. Even though the usage of mobile phones in hospitals has a lot of benefits, it is still a large source of contamination. This is because when in use, mobile phones generate heat, which provides suitable conditions for the replication of bacteria present on them. In a 2012 study in Croatia, samples were collected from 50 and 60 mobile phones from HCW and 60 medical students, respectively. Out of the 110 mobile phones about 35% were contaminated with one type of microorganism, 28% with two types and 15% with three types. According to the results, the most commonly isolated microorganisms were coagulase negative staphylococci and *Staphylococcus aureus* (*S. aureus*) (26%) [2]. The second most abundant bacteria were *S. aureus* with 50% of the colonies being methicillin-resistant [4]. Normally, *S. aureus* is found as a commensal bacterium on the skin and in the upper respiratory tract. A wide range of infections can be caused by pathogenic strains of *S. aureus* [5]. Most frequently it causes skin infections, which can manifest in different forms such as boils, folliculitis, cellulitis or even more invasive soft tissue infections. Due to the vastly changing nature of *S. aureus*, a lot of research is needed, particularly in the field of nosocomial infections [6]. The most researched strain is methicillin-resistant *S. aureus* (MRSA), which has become a global health concern over the last couple of years [7]. Infections caused by these multi-drug resistant gram-positive organisms are a worldwide problem in hospitals and are a general health hazard to the population since they require a specific type of antibiotic for treatment. The purpose of this study was to isolate *S. aureus* from mobile devices of undergraduate students from the University Colleges Leuven-Limburg, and UZIMA University and determine the antimicrobial sensitivity patterns of isolated pathogens.

## Main text

Most microorganisms found in this study, were gram-positive bacteria that can easily survive on or are transferred by hands. Bacteria such as *S. aureus* isolated on mobile devices can survive for a long period in a dehydrated environment such as the skin of the hands, the surface on mobile phones and medical devices.

### Methods

This research was done at UZIMA University after obtaining approval from the Institutional Review Board. UZIMA University is located in Kisumu County, in Western Kenya. The University offers undergraduate courses in various disciplines such as Medicine, Nursing, Clinical medicine, Microbiology, Health records and Community health.

An experimental study design was adopted where a total of 16 mobile phones were randomly selected from students. The primary outcome was to isolate *Staphylococcus aureus* from surfaces of mobile phones. These phones were obtained from students of Microbiology and Biomedical Laboratory Sciences from UZIMA University, Kisumu (Kenya) and the University Colleges Leuven- Limburg, Leuven (Belgium), respectively. The research was part of an annual international project between the two academic institutions to enhance student linkages and experience on practical aspects in the field of microbiology.

Swabs from surfaces of mobile phones were collected using disposable sterile cotton swabs moistened with sterile normal saline.

Approximately 1.84 g of nutrient agar (Fisher Scientific, Leicestershire, UK) was weighed and dissolved in 80 ml of distilled water in a glass beaker. The glass beaker was heated until the agar was completely dissolved. The media was sterilized by autoclaving at 121 °C for 15 min. Mobile swabs were plated onto nutrient agar and incubated at 37 °C for 24 h. After incubation, the colonies were identified by their characteristic appearance on nutrient agar.

Gram staining was used for differentiation of gram-positive and gram-negative bacteria by microscopy. Smears were made on microscopic slides from the colonies. The smears were air-dried and heat fixed and flooded with crystal violet (Fisher Scientific, Leicestershire, UK) staining reagent for 1 min. The slides were then washed gently in running water for 2 s. The slides were then flooded with gram’s iodine (Fisher Scientific, Leicestershire, UK) for 1 min and thereafter washed in a gentle and indirect stream of tap water for 2 s. The slides were decolorized for 15 s with acetone (Fisher Scientific, Leicestershire, UK) and counterstained with safranin (Fisher Scientific, Leicestershire, UK) for 1 min. The slides were finally washed in running water and blotted on an adsorbent filter paper.

The catalase method was used to differentiate *Staphylococci* from *Streptococci* bacteria. Briefly, a small amount of bacterial colony was added to a glass using a sterile loop. A drop of 3% H_2_O_2_ (Fisher Scientific, Leicestershire, UK) was then added onto the slide and mixed with the colony.

The coagulase method was used as a confirmatory test for *Staphylococcus aureus*. A drop of staphylococcal colony was emulsified on a glass slide. A sterile wire loop was dipped into undiluted plasma and then emulsified onto the staphylococcal suspension. Coarse clumping was confirmatory for *Staphylococci aureus.*

The Kirby–Bauer disc diffusion susceptibility test method was used to determine the sensitivity or resistance of bacteria to antibiotics. Mueller–Hinton agar (Fisher Scientific, Leicestershire, UK) was prepared by weighing 1.84 g of agar in 80 ml of distilled water. The media was briefly heated on a Bunsen burner flame to dissolve and autoclaved as previously described. The media was then dispensed on agar plates and left to solidify. An inoculum was prepared as follows: A sterile inoculating loop was used to pick colonies of the test organism and suspended in 2 ml sterile saline in a tube. A sterile swab was dipped into the inoculum tube and rotated against the side of the tube. The dried surface of the Mueller–Hinton agar plate was inoculated by streaking the swab three times over the entire surface. The antimicrobial impregnated disks (Sigma-Aldrich, Missouri, USA) were placed on the surface of the agar plates using a pair of sterile forceps. The plates were placed inverted in an incubator for 24 h. The zones of inhibition were measured in mm and the results were scored as resistant, intermediate or susceptible based on the Clinical Laboratory Standards Institute Performance Standards for Antimicrobial Disk Susceptibility Tests.

### Results

**Fig. 1:**
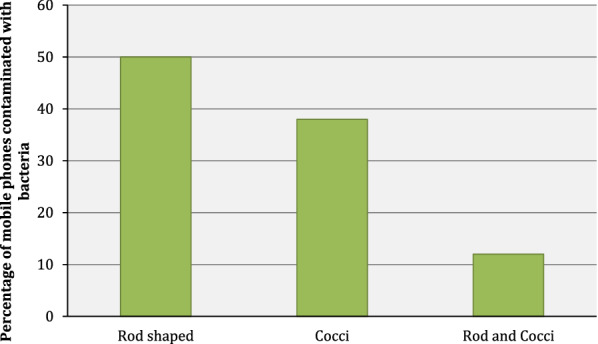
The morphological forms isolated from mobile phones used in this study were classified into three categories, rods, cocci and both rod and cocci

Gram-positive bacteria were isolated from all the mobile phones used in this study (n = 16). These bacteria were grouped into three groups according to the morphological features after gram staining procedure, rod shaped (n = 8), cocci (n = 6) and mixed (n = 2) (rod and cocci) as shown in Fig. 1.

Only the cocci and the mixed species were further subjected to the catalase test (n = 8) to identify the Staphylococci species. All the samples tested were catalase positive.

**Fig. 2:**
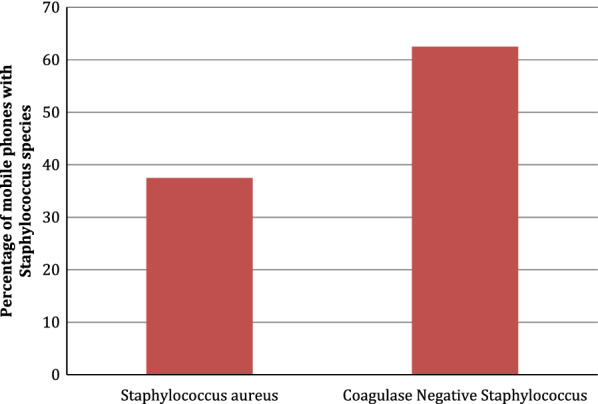
The coagulase test was used to identify *Staphylococcus aureus* from coagulase negative *Staphylococcus*

Coagulase negative *Staphylococcus species* were isolated in 5/8 (62.5%) and 3/8 (37.5%) mobile devices had *Staphylococcus aureus* as shown in Fig. 2.

**Fig. 3:**
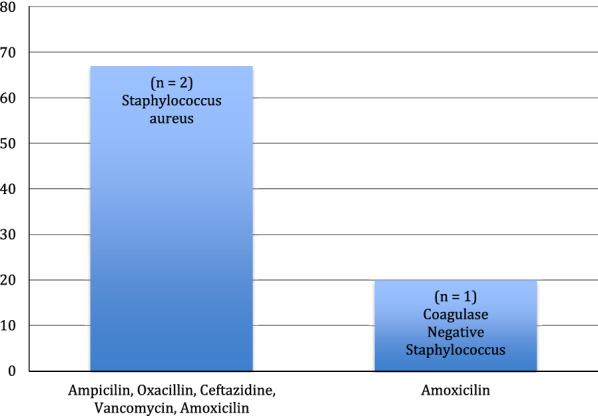
*Staphylococcus aureus* isolates from two mobile devices was resistant to ampicillin, oxacillin, ceftazidine, vancomycin and amoxicillin whereas only one Coagulase negative isolate was resistant amoxicillin

Two out of three (67%) *Staphylococcus aureus* strains were resistant to the following antibiotics; ampicillin, oxacillin, ceftazidime, vancomycin and amoxicillin and 1/5 (20%) coagulase negative streptococcus was resistant to amoxicillin as shown in Fig. 3.

### Discussion

Globally, there are increasing cases of bacterial infection in humans. This is mainly attributed to factors such as contamination of surfaces of mobile phones that eventually become fomites spreading pathogenic organisms particularly by hands.

Studies on the extent of bacterial colonization of mobile phones will ensure adoption of preventive measures such as disinfection procedures, establishing of mobile phone free zones in hospitals, designing replaceable devices that contain less bacterial contamination and making people aware of the current situation about AMR. Once instituted, these measures will reduce the emergence of antimicrobial resistant isolates and nosocomial infections.

All phones used in this study showed significant microbial growth. Our research reported 50% (8/16), gram-positive rod shaped bacteria on mobile phones. This observation is similar to a study that was done in New Zealand at the Fiji National University. In this study, 82% of mobile phones that were sampled had gram-positive bacillus bacteria [5]. Studies have confirmed that spore-forming gram-positive rods, such as Clostridia, present on mobile devices can be transferred into human food and cause illness once ingested [6]. Our findings, however, are in contrast to a previous study in Slovakia that investigated the effectiveness of mobile phone disinfection in the microbiology laboratory [7]. In this study, 36% of mobile phones sampled were contaminated with Bacillus bacteria [7]. The reason for this discrepancy could be in the varying sample sizes between the Slovakian study and our research. Comparing the results between the Belgian phones and the Kenyan phones is difficult, because only one sample of *S. aureus* from each country was used in antimicrobial susceptibility test. This is not enough to make a correct conclusion about the difference in resistance between Kenya and Belgium.

In this research, Coagulase negative Staphylococcus (CoNS) and *S. aureus* accounted for 62.5% (5/8) and 37.5% (3/8) of the gram-positive cocci bacteria present on the mobile phones, respectively. A recent meta-analysis review in 2020 on previous studies on common bacterial isolates from mobile phones of health care workers confirmed that 66% of mobile phones were contaminated with CoNS [8]. The same paper also reported that 91% of the phones were colonized with *S. aureus* [8]. CoNS are commensals of the human skin and mucosal surfaces and cause infections mostly in neonates and immunocompromised individuals [9]. Species include *S. hominis, S. capitis, S. warneri*, *S. epidermidis,* and *S. haemolyticus. S. epidermidis* is the normal microbiota of the nasal surfaces and the newborn umbilicus [9]. *S. aureus* is an opportunistic bacteria, responsible for nosocomial and community infections [10]. In addition, *S. aureus* can invade tissues and cause infections such as cutaneous abscesses, endocarditis, and septic shock [10].

Our findings showed that 67% of the *S. aureus* isolates were resistant to ampicillin, oxacillin, ceftazidine, vancomycin and amoxicillin. A previous study in Nigeria revealed that 42.8% of *S. aureus* isolated from mobile phones of non-health care workers was resistant to ampicillin [11]. Clinically, OXACILLIN is used as a marker to screen for *mec-A-*mediated resistance in *Staphylococcus* species*,* therefore, methicillin-resistant isolates are usually identified in culture by using oxacillin break points [12]. Compared to oxacillin susceptible strains, resistant *S. aureus* strains are slow growers and often show heteroresistance [12]. A past research study reported that methicillin resistant prevalence of *S. aureus* isolated from mobile phones of medical and non-medical staff working in the emergency section of the hospital was 12% [13]. In Bangladesh, research focused on the presence of multidrug-resistant bacteria on mobile phones of health workers reported that 50% of the *S. aureus* isolates were resistant to Ceftazidine [14]. In a study in Saudi Arabia, bacteria was isolated from mobile phones of health care workers and tested against a panel of 12 antimicrobial agents, only one *S. aureus* isolate had Vancomycin heteroresistance after sensitivity testing [15]. The same study also reported that 38.6% of the cell phones had been colonized by CoNS. Our research also showed that only one CoNS isolate was resistance to amoxicillin. A meta-analysis research that analyzed 45 studies on the resistance of *S. aureus* in Ethiopia showed that 77% of *S. aureus* were resistant to amoxicillin [16]. There was a higher level of AMR present in the Kenyan sample. The Belgian sample shows a higher susceptibility to penicillin based antimicrobials whereas the Kenyan sample shows complete resistance.

In this study, the most abundant bacteria were the coagulase-negative staphylococci with five colonies and *S. aureus* was present on three mobile phones. Two *S. aureus* (one from Kenya and one from Belgium) showed high resistance against a large number of antimicrobials. Additional research on a larger scale is needed for a more in depth analysis. Altogether, the contamination indicates the potential risk of nosocomial infections in hospital environments and indicates the need of measures for the utilization of mobile phone in the health care sector and hand hygiene after usage, to prevent the spread of resistant strains.

## Limitations

Findings from our research should be interpreted with caution because of the small number of mobile phones used. In addition, our research only focused on two types of bacterial species.

## Supplementary Information

**Additional file 1:** **Table S1.** Results from gram staining, catalase and coagulase test and the difference between Kenyan and Belgian students. **Table S2.** Results antimicrobial susceptibility test: inhibition zone comparing standard zone.

## Data Availability

All data generated or analyzed during this study are included in this published article (Additional file 1).

## Abbreviations

AMR: Antimicrobial resistance
CoNS: Coagulase negative *Staphylococcus*
HCW: Health care workers
MRSA: Methicillin-resistant *S. aureus*
