# Supplementary material for: Antimicrobial sensitivity patterns of Staphylococcus species isolated from mobile phones and implications in the health sector
Source: BMC Res Notes. 2021 Jan 6;14:1. doi: 10.1186/s13104-020-05413-7 (PMC7788780; doi:10.1186/s13104-020-05413-7)
Supplement: Supplementary file 1 — Additional file 1: Table S1. Results from gram staining, catalase and coagulase test and the difference between Kenyan and Belgian students. Table S2. Results antimicrobial susceptibility test: inhibition zone comparing standard zone. [file 13104_2020_5413_MOESM1_ESM.docx]

*Table S1: Results from gram staining, catalase and coagulase test and the difference between Kenyan and Belgian students*

| Student | Nationality | Gram | +/- | Shape | Catalase | Coagulase |
| --- | --- | --- | --- | --- | --- | --- |
| 1 | Belgium | + | Rods | | - |  |
| 2 | Kenya | + | Cocci | | + | - |
| 3 | Belgium | + | Rods | | + |  |
| 4 | Belgium | + | Rods | | - |  |
| 5 | Kenya | + | Rods | | + |  |
| 6 | Belgium | + | Rods | | + |  |
| 7 | Belgium | + | Cocci/rods | | + | + |
| 8 | Kenya | + | Rods | | - |  |
| 9 | Belgium | + | Cocci | | + | - |
| 10 | Kenya | + | Rods | | + |  |
| 11 | Kenya | + | Cocci/rods | | + | + |
| 12 | Belgium | + | Cocci | | + | - |
| 13 | Kenya | + | Cocci | | + | - |
| 14 | Kenya | + | Cocci | | + | - |
| 15 | Kenya | + | Rods | | - |  |
| 16 | Belgium | + | Cocci | | + | + |

*Table S2: Results antimicrobial susceptibility test: inhibition zone comparing standard zone.*

Antimicrobial Standard zone

(mm)

Inhibition zone (mm)

Results

| Sample Belgian | AMP | 27-35 | 8 | Resistant |
| --- | --- | --- | --- | --- |
| *S. aureus* | OX | 18-24 | 0 | Resistant |
|  | COT | 24-32 | 16 | Resistant |
|  | CAZ | 16-20 | 9 | Resistant |
|  | DO | 23-29 | 7 | Resistant |
|  | E | 22-30 | 5 | Resistant |
|  | VA | 17-21 | 0 | Resistant |
|  | AMC | 28-36 | 6 | Resistant |
| Sample Kenyan *S. aureus* | AMP  OX | 27-35  18-24 | 0  0 | Resistant  Resistant |
|  | COT | 24-32 | 11 | Resistant |
|  | CAZ | 16-20 | 0 | Resistant |
|  | DO | 23-29 | 15 | Resistant |
|  | E | 22-30 | 5 | Resistant |
|  | VA | 17-21 | 10 | Resistant |
|  | AMC | 28-36 | 0 | Resistant |
| Sample Coagulase - | AMP | 27-35 | 20 | Resistant |
| staphylococci | OX | 18-24 | 10 | Resistant |
|  | COT | 24-32 | 11 | Resistant |
|  | CAZ | 16-20 | 11 | Resistant |
|  | DO | 23-29 | 14 | Resistant |
|  | E | 22-30 | 17 | Resistant |
|  | VA | 17-21 | 10 | Resistant |
|  | AMPC | 28-36 | 0 | Resistant |

| Sample gram-positive catalase-positive bacillus | AMP  OX | 27-35  18-24 | 8  7 | Resistant  Resistant |
| --- | --- | --- | --- | --- |
|  | COT | 24-32 | 15 | Resistant |
|  | CAZ | 16-20 | 0 | Resistant |
|  | DO | 23-29 | 15 | Resistant |
|  | E | 22-30 | 8 | Resistant |
|  | VA | 17-21 | 1 | Resistant |
|  | AMX | 28-36 | 0 | Resistant |

*AMP: ampicillin; OX: oxacillin; COT: co-trimoxazole; CAZ: ceftazidime; DO: doxycycline Hcl; E: erythromycin; VA: vancomycin; AMC: amoxyclav*
